# The protective efficacy of inactivated vaccine against hemorrhagic fever with renal syndrome: A meta-analysis: Erratum

**DOI:** 10.1097/MD.0000000000050151

**Published:** 2026-08-14

**Authors:** Weiting Wu, Jie Liu, Shicheng Guo, Han Zhao, Xuli Yang

In the article “The protective efficacy of inactivated vaccine against hemorrhagic fever with renal syndrome: A meta-analysis”,^[1]^ which appears in Volume 104, Issue 20 of *Medicine*, the text changes have been made in three sections of the article. (1) The number “ten” has been changed to “thirteen” in section (3.1. Selection of study), second paragraph, first sentence: “Of the 15 [12,17-30] articles included, only two were foreign articles, and both were from South Korea, while the remaining ten were from China;” (2) In 3.4 sub-section, the second sentence “The protection rate of HFRS inactivated vaccine in China was 86% (95% CI: 70%–93%)” has been deleted; and (3) In the Discussion section, in final paragraph: ‘not’ deleted from the first sentence “The inactivated HFRS vaccine has not been included in the national immunization program in China”.

## References

[1] Wu W Liu J Guo S Zhao H Yang X. The protective efficacy of inactivated vaccine against hemorrhagic fever with renal syndrome: a meta-analysis. Medicine (Baltimore). 2025;104:e42463. doi: 10.1097/MD.0000000000042463. 40388733 10.1097/MD.0000000000042463 PMC12091670

